# NK Cells: Uncertain Allies against Malaria

**DOI:** 10.3389/fimmu.2017.00212

**Published:** 2017-03-09

**Authors:** Asia-Sophia Wolf, Samuel Sherratt, Eleanor M. Riley

**Affiliations:** ^1^Department of Immunology and Infection, London School of Hygiene and Tropical Medicine, London, UK

**Keywords:** NK cells, malaria, inflammatory cytokines, cytotoxic killing, KIR

## Abstract

Until recently, studies of natural killer (NK) cells in infection have focused almost entirely on their role in viral infections. However, there is an increasing awareness of the potential for NK cells to contribute to the control of a wider range of pathogens, including intracellular parasites such as *Plasmodium* spp. Given the high prevalence of parasitic diseases in the developing world and the devastating effects these pathogens have on large numbers of vulnerable people, investigating interactions between NK cells and parasitized host cells presents the opportunity to reveal novel immunological mechanisms with the potential to aid efforts to eradicate these diseases. The capacity of NK cells to produce inflammatory cytokines early after malaria infection, as well as a possible role in direct cytotoxic killing of malaria-infected cells, suggests a beneficial impact of NK cells in this disease. However, NK cells may also contribute to overproduction of pro-inflammatory cytokines and the consequent immunopathology. As comparatively little is known about the role of NK cells later in the course of infection, and growing evidence suggests that heterogeneity in NK cell responses to malaria may be influenced by KIR/HLA interactions, a better understanding of the mechanisms by which NK cells might directly interact with parasitized cells may reveal a new role for these cells in the course of malaria infection.

## Introduction

Natural killer (NK) cells are a subset of lymphocytes that contribute to the control of cancers and infections through the production of pro-inflammatory cytokines and the destruction of damaged, dysfunctional or infected host cells *via* cytotoxic activity [reviewed in Ref. (1)]. They typically constitute about 10% of peripheral blood mononuclear cells (PBMCs), although there is considerable variation between individuals. The activity of NK cells is regulated by binding of antibody–antigen complexes to the Fc receptor CD16 (2), expression of a large range of activating and inhibitory receptors used to directly “read” the surface of potentially infected or dysfunctional cells [reviewed in Ref. (3, 4)], and expression of receptors for cytokines such as interleukin (IL)-12, IL-15, IL-18 and IL-2 [reviewed in Ref. (5)]. Healthy cells express ligands for inhibitory NK cell receptors, ensuring that they are “ignored” by patrolling NK cells, but these ligands are downregulated on damaged or diseased cells, while activating signals (so-called “stress ligands”) may be upregulated, making the cells clear targets for NK cell-mediated destruction. Moreover, pro-inflammatory cytokines can override ligand-mediated inhibitory signals, thereby allowing NK cells to participate in systemic immune responses by producing inflammatory cytokines (6–8).

Although traditionally classed as innate lymphocytes, recent work has suggested that NK cells may participate in adaptive immune responses and may also exhibit immunological “memory” or “memory-like” responses leading to significantly higher cytokine production and enhanced cytotoxic responses upon restimulation. This topic was recently comprehensively reviewed by Cerwenka and Lanier (9), but, in brief, enhanced NK cell responses have been described after infection with viruses, after exposure to haptens, and after *in vitro* stimulation with cytokines. Very recently, enhanced responses of human peripheral blood NK cells have also been observed *ex vivo* after influenza vaccination (10). While there is some evidence in murine systems, and more recently in rhesus macaques (11), that these “memory” NK cell responses may be antigen specific, this has only been shown definitively for liver-resident NK cells (12, 13) and the only well-characterized receptor–ligand interaction is the mouse Ly49 receptor family binding murine cytomegalovirus (MCMV) ligands (14–17). In the case of human CMV (HCMV), the functionally equivalent interaction is mediated by heterodimeric CD94/NKG2A and CD94/NKG2C receptors which recognize peptides from HCMV bound to human leukocyte antigen (HLA)-E (18) and which induce characteristic expansions of the NKG2C^+^ NK cell subset and epigenetic modifications of the NK cell genome (19–22) [reviewed in Ref. (23)]. However, in many cases such as in studies on malaria, rabies, and influenza, these enhanced secondary responses are at least partly attributable to indirect activation of NK cells by memory T cell-derived IL-2 rather than to true “memory” on the part of NK cells themselves (10, 24–26). This proxy recall response was first identified during influenza vaccination by He et al. (27) and then by Horowitz et al. (24) in response to rabies vaccination. Subsequent studies have demonstrated a similar IL-2-dependent effect in response to malaria-infected erythrocytes (25). Regardless of the underlying mechanism, this raises the intriguing possibility that NK cells may contribute substantially to immune responses after malaria vaccination, and preliminary studies have already demonstrated enhanced NK cell activation in response to increased T cell IL-2 production in individuals vaccinated with the RTS,S/AS01 malaria vaccine (26).

Given this evidence, there is considerable interest in gaining a better understanding of the mechanisms by which NK cells are activated during malaria infections and whether this is beneficial or detrimental. Such research will serve to clarify the basic functions of NK cells during infection with intracellular protozoa and, potentially, to target an effective immune mechanism during vaccine development. In this review, we summarize the current state of knowledge of the role of NK cells during malaria infection and malaria vaccination, both in humans and in experimental murine infections.

## Mechanisms of NK Cell Activation

Natural killer cells were classically considered “natural” killers because, unlike T cells, they do not require prior exposure to antigen before being able to engage and kill target cells, although it is now understood that they require a complex process of education and licensing in order to become fully functional (7, 28). During infection, the main functions of NK cells are cytokine production and cytotoxic killing of infected host cells. These activities can be triggered by three distinct but complementary activation pathways: cytokine activation, antibody-dependent cell-mediated cytotoxicity (ADCC), and loss of inhibitory signaling due to downregulation or mismatching of major histocompatibility complex (MHC) class I (the missing-self hypothesis). NK cells can be activated *via* a plethora of host (target) cell surface receptors, including activating members of the killer cell immunoglobulin-like receptor (KIR) family that bind to MHC molecules [reviewed in Ref. (29)], killer lectin-like receptors (KLRs) such as NKG2D homodimers and CD94/NKG2A and CD94/NKG2C heterodimers that interact with HLA-E, and natural cytotoxicity receptors (NCRs) such as NKp30 and NKp46 which are believed to recognize pathogen-encoded ligands [reviewed in Ref. (30)]. The outcome of these interactions can be direct lysis of the target cell by the NK cell, which implies an important role for NK cells in killing infected or diseased cells. NK cells also constitutively express receptor subunits for IL-15, IL-18, and IL-12, as well as the low-affinity receptor for IL-2. The high-affinity IL-2 receptor α chain (CD25) is upregulated upon activation, allowing NK cells to become activated by cytokines as a result of local or systemic inflammation (8, 31).

Natural killer cells are also significant producers of inflammatory cytokines during early infection, prior to the priming, clonal expansion and activation of antigen-specific T cells (32–35); in particular, NK cells are an important source of interferon gamma (IFN-γ). *In vitro*, NK cells are capable of producing IFN-γ after activation by exogenous cytokines, in particular IL-12 and IL-18 (8, 31, 36, 37). However, during infection, various cytokines are produced at different times from diverse cellular sources, allowing NK cell responses to be finely controlled so that pathogen growth and dissemination is constrained while simultaneously limiting the pathology caused by uncontrolled inflammation (38–40). For example, during infection with two classic Th1-inducing pathogens, *Salmonella enterica* and *Plasmodium falciparum*, NK cells require activating signals from three distinct sources: a priming or pre-activation signal of either IL-2 or IL-15 from T cells or dendritic cells (DCs) respectively, IL-12 and IL-18 from macrophages, and direct contact with macrophages (41, 42). Activation *via* these pathways allows synthesis of IFN-γ by NK cells that induces downstream transcription of IFN-controlled genes, thereby leading to macrophage activation and killing of target cells or pathogens.

## Malaria Symptoms and Spread

**Figure 1:**
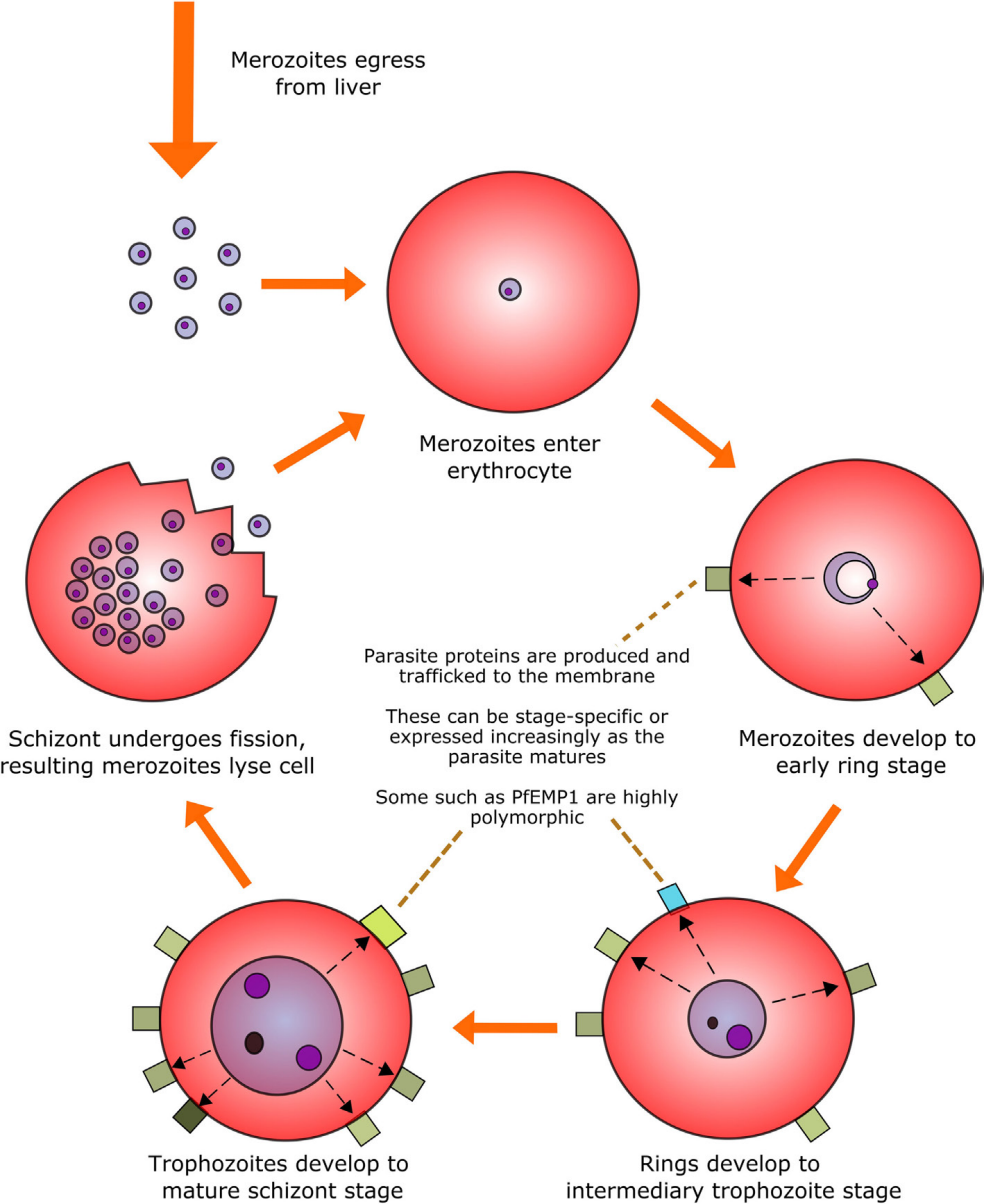
**The intra-erythrocytic asexual replication cycle of malaria parasites**. After initial infection of the host and replication in the liver, merozoites are released into the blood stream where they penetrate healthy erythrocytes. The merozoite will then metabolize host hemoglobin to fuel its own development into the mature schizont stage (over the course of approximately 48 h in the case of *P. falciparum*) while restructuring the erythrocytic membrane to aid in nutrient transfer, rosetting and sequestration, and producing proteins, some of which are exported to the erythrocyte surface. The mature schizont will then undergo replicative fission, forming 8–32 merozoites which lyse the cell membrane and re-enter the bloodstream.

Perhaps the most infamous family of protozoan parasites affecting humans is *Plasmodium*, the causative agent of malaria in a range of mammalian and non-mammalian hosts. The parasites are carried by numerous species of mosquito vectors of the *Anopheles* genus and are transmitted by female mosquitoes during hematophagy. During a single bite, between a dozen and several hundred motile and infectious malaria sporozoites are inoculated into the skin of the mammalian host from the salivary glands of the mosquito; they then disseminate to the liver *via* the blood stream to initiate the intravertebrate developmental pathway (43–45). The developmental pathway of the *Plasmodium* parasite within its vertebrate host comprises multiple life stages exhibiting both constitutive and stage-specific genomic and proteomic expression (46). Of particular clinical relevance is the intra-erythrocytic stage during which merozoites enter erythrocytes and develop into trophozoites and large, multinucleated schizonts. These schizonts then lyse their host cells in order to release the next generation of merozoites into the circulation and repeat the hematic replication cycle (Figure 1).

In humans, this intra-erythrocytic stage of the parasite life cycle causes the symptoms of malaria infection. The lysis of erythrocytes can lead to severe anemia, and structural changes induced in the red blood cell membrane by the parasite can lead to vascular sequestration (i.e., adherence of parasitized erythrocytes to vascular endothelium, causing blockage of small blood vessels). Anemia and sequestration can in turn lead to systemic lactic acidosis due to reduced oxygen delivery to the tissues, and in the brain can lead to cerebral malaria, resulting in seizures, coma, and potentially death [reviewed in Ref. (47)]. Excessive inflammatory immune responses, characterized as high levels of IL-1, IL-6, IFN-γ, and TNFα, exacerbate these direct effects of parasitemia, leading to immunopathology (39, 48–51). However, an insufficient inflammatory response is conversely associated with increased parasitemia and poorer outcomes (39, 40), indicating that an optimal immunological response to malaria is a delicate balancing act. The nature of this paradox will be discussed in detail later in this review.

It is estimated that 3.2 billion people are currently at risk of developing malaria, and in 2015 alone there were an estimated 212 million cases of malaria leading to approximately 429,000 deaths worldwide; of these deaths, 70% were in children under 5 years old (52). The burden of malaria falls most heavily on Sub-Saharan Africa and South Asia; 80% of cases and 90% of deaths occurred in Africa, with the Democratic Republic of the Congo and Nigeria alone accounting for an estimated 35% of global malaria mortality. Current methods for controlling the spread of malaria include the use of long-lasting insecticide-treated bed nets, residual indoor spraying with insecticides, chemoprevention in vulnerable individuals, and combination drug treatment of infected individuals (52). While these tools have curbed the transmission of malaria in the last decades, it is widely accepted by both researchers and public health officials that sustainable malaria control or elimination would be facilitated by a highly effective malaria vaccine, the development of which requires a greater scientific understanding of the interaction between the human immune system and *Plasmodium* parasites.

There are currently six species of *Plasmodium* that are known to cause malaria in humans (53), in addition to three capable of causing similar symptoms in mice [reviewed in Ref. (54)] (Table 1). Of the six species capable of infecting humans, *P. falciparum* and *P. vivax* are responsible for most malaria deaths worldwide*. P. falciparum* is responsible for most malaria deaths in Africa (and therefore most global deaths due to Africa’s disproportionate malarial burden) and is the most studied human strain of malaria, which is greatly assisted by its tolerance for *in vitro* laboratory culture. By contrast, *P. vivax* has so far been unamenable to long-term laboratory culture. This is a major impediment to further understanding of this species, contributing to its continued status as a major health burden across Asia and South America.

**Table 1:** **List of *Plasmodium* species causing malaria infection in mice or humans**.

Species	Common mammalian host(s)
*Plasmodium falciparum*	*Homo sapiens*
*Plasmodium vivax*	*H. sapiens*
*Plasmodium malariae*	*H. sapiens*
*Plasmodium ovale curtisi*	*H. sapiens*
*Plasmodium ovale wallikeri*	*H. sapiens*
*Plasmodium knowlesi*	*H. sapiens*/*Macaca fascicularis*
*Plasmodium chabaudi*	Various *Rodentia* species including murids
*Plasmodium berghei*	Various *Rodentia* species including murids
*Plasmodium yoelii*	Various *Rodentia* species including murids

## Malaria in Mice and the Importance of NK Cells

A rapid and robust pro-inflammatory immune response is essential for control of malaria parasitemia. Much of the research underpinning this observation has been performed in mice infected with species of *Plasmodium* that naturally infect wild rodents, and has shown a crucial role for IFN-γ in parasite control and clearance (55–57) [reviewed in Ref. (58)] and a decreased likelihood of mice developing the severe symptoms of cerebral malaria (59). It is worth noting, however, that the effects of IFN-γ during *Plasmodium* infection vary depending on the amount produced, the time course of cytokine production, and the particular characteristics of the *Plasmodium* strain involved (Table 2). IFN-γ is a crucial mediator of antimalarial effector mechanisms and is thought to act primarily by activating macrophages to phagocytose merozoites and parasitized erythrocytes in both an opsonization-dependent (60) and opsonization-independent manner (61), and by inducing macrophages to produce parasiticidal free radicals such as nitric oxide (NO) and superoxides, which combine to form short-lived but highly damaging peroxynitrite capable of efficiently killing infected erythrocytes (62).

**Table 2:** **List of experimental *Plasmodium* infection models in mice**.

Species	Strain	Characteristics
*Plasmodium yoelii*	Py17X	Non-lethal, self-resolving. Also known as PyNL or PyXNL
	Py17XL	Lethal. Also known as PyL
	PyYM	Lethal
*Plasmodium berghei*	ANKA	Lethal. Causes experimental cerebral malaria in “susceptible” C57BL/6 mice; “resistant” BALB/c mice subsequently die of severe anemia
	NK65	Lethal. Does not cause cerebral malaria
*Plasmodium chabaudi*	AS	Non-lethal in C57BL/6 mice, lethal in “susceptible” A/J mice

Given the capacity of NK cells to secrete large amounts of IFN-γ very quickly (63, 64), it is reasonable to assume that NK cells may contribute to control of malaria infections, and indeed several studies have demonstrated a crucial role for NK cells in the production of cytokines during murine malaria infections (55, 65). Murine splenic, hepatic and peripheral blood NK cells have been shown to significantly upregulate their production of pro-inflammatory cytokines such as IFN-γ and TNFα in response to both erythrocytic and hepatic stages of *Plasmodium yoelii* (55, 66), as well as blood-stage *Plasmodium chabaudi* (55, 65). It has also been demonstrated that experimental depletion of NK cells in mice infected with *P. yoelii* or *P. chabaudi* results in a decrease in IFN-γ production with a corresponding increase in parasitemia (57, 65), suggesting that NK cells contribute significantly to the early production of pro-inflammatory cytokines that is associated with an improved clinical outcome. Additionally, NK cells play an important role in reciprocal activation of DCs for cytokine production and CD4 T cell priming during murine malaria infections (67, 68), placing them at the interface of innate and adaptive immunity.

In pre-erythrocytic stages of infection, IFN-γ produced by proliferating hepatic NK cells inhibits the growth of hepatic schizonts (66, 69). Type I interferons (IFN-α and -β) produced by plasmacytoid DCs (pDCs) are thought to be important drivers of hepatic NK cell activation (69–71) as mice deficient in IFNAR (the IFN-α/β receptor) were unable to reduce the burden of liver-stage parasites in *P. yoelii* non-lethal infections (69, 72). However, some studies have suggested that NK T cells and/or non-conventional γδ T cells may play a greater role than NK cells in driving the early cytokine-driven inflammatory response (69, 73). This uncertainty may be partly due to variability in the time points of infection analyzed, as well as differences between strains of *Plasmodium* infection and murine models. In a dynamic system such as the immune system, with high levels of redundancy, it is likely that more than one cell type or mechanism of protection contributes to the outcome of infection.

The role of IL-15 in priming NK cells has not been extensively studied in mouse models of malaria, but work by Ing et al. indicates that DC-derived IL-15 enhances NK cell production of IFN-γ in combination with IL-12 (67, 74); this is consistent with *in vitro* studies of human NK cells showing that IL-15 is an important priming signal for NK cell activation (38), and that combinations of cytokines are required to drive IFN-γ production by NK cells (8, 31, 32, 75). IL-12 is a key driver of IFN-γ production (31, 33, 76), and loss of IL-12 results in decreased IFN-γ responses, higher parasitemia, and less effective malaria-specific antibody responses (77, 78). In tandem with this, IL-2 signaling is thought to promote full activation of NK cells (based on human *in vitro* studies by Horowitz et al. (63)). For many years, it was believed that CD25, the high-affinity IL-2 receptor subunit, was not expressed on murine NK cells (79) and reports on murine malaria infections tended to support this view (66), but more recent work has found a clear role for CD25 expression, primarily driven by IL-18 but further enhanced by IL-12, during murine malaria infections (64) and MCMV infection (80). Furthermore, there appears to be a strong correlation between CD25 expression and IFN-γ production, which supports a role for T cell-derived IL-2 in maintaining or driving NK cell responses in both humans and mice (63, 64). The capacity to respond to T cell-mediated signals may also indicate a role for NK cells beyond early infection.

In murine models of virulent malaria infections, excessive IFN-γ or TNF production can lead to severe immunopathology (81–84) suggesting that, although NK cells are beneficial during early immune responses to malaria, they may contribute to the detrimental effects of excessive systemic inflammation (83). There is also evidence that NK cells may recruit T cells to the brain during murine *Plasmodium berghei* infections and therefore contribute to the development of experimental cerebral malaria (83, 85). In keeping with this, the anti-inflammatory cytokines IL-10 and TGF-β have been shown to regulate the pro-inflammatory immune response during malaria infection, counteracting the pathological effects of inflammatory cytokines and promoting healthy resolution of the immune response after initial stimulation (86–88). Neutralization of TGF-β is lethal in a normally non-lethal *P. chabaudi chabaudi* infection (88), and lack of IL-10 in IL-10^−/−^ mice leads to increased IFN-γ, TNFα and IL-12 production and exacerbated pathology and mortality (86, 89). IL-10 and TGF-β appear to show some overlap in function and can individually downregulate pro-inflammatory responses (87), although both are suggested to modulate macrophage activation (89, 90). The regulatory receptors CTLA-4 and PD-1 are also thought to be important regulators of inflammation and are frequently co-expressed on activated T cells during infection; blockade of either receptor has been shown to induce lethal cerebral malaria in normally resistant BALB/c mice (91). However, overexpression of TGF-β or IL-10 very early in infection inhibits the pro-inflammatory response and impedes parasite clearance (89). Similarly, blockade of CTLA-4 drives excessive inflammation and exacerbates pathology in mice infected with a lethal strain of *P. yoelii*, but mediates lower peak parasitemia and swifter parasite clearance in a non-lethal model (81). There is some evidence in other disease models, including from other protozoan infections, for NK cells as producers of IL-10 (92–95), drawing parallels with CD4 T cells that can produce both IFN-γ and IL-10. To date, IL-10-producing NK cells have not been reported in the context of malaria exposure or infection, but it is certainly possible that “regulatory” NK cells might be found to contribute to healthy resolution of the inflammation associated with malaria infections.

## NK Cells and Cytokine Responses to Malaria in Humans

Evidence that NK cells contribute to the antimalarial immune response in experimental murine models has naturally provoked interest in establishing whether the same is true for human *Plasmodium* infections. As anticipated from experimental animal studies, numerous studies conducted on human populations have revealed positive associations between IFN-γ production and protection against malaria infection [reviewed in Ref. (96)]. IFN-γ production by PBMCs has been found to be associated with mild rather than severe malaria in children, and children with mild malaria who had detectable IFN-γ responses also demonstrated delayed incidence of reinfection within 1 year of initial infection (97). More recently, long-term protection against experimental malaria infection after vaccination with whole *P. falciparum* sporozoites while under protective antimalarial drug cover (98) has been associated with both IFN-γ and T cell IL-2 production by PBMCs restimulated *in vitro* with sporozoite antigen (99).

However, as previously demonstrated in mice, excessive pro-inflammatory cytokine production is also associated with onset of clinical disease and immunopathology in humans. An early study in African children by Riley et al. in 1991 demonstrated an association between IFN-γ production after *in vitro* stimulation with malaria antigens and an increased likelihood of developing fever and malaise during *in vivo* infection (49). A later study by Walther et al. found an association between the presence of IFN-γ in plasma in the first few days after experimental malaria infection and fever (39). Other studies have suggested that IFN-γ, TNFα, and IL-12 production by PBMCs are associated with lower parasite densities and higher hemoglobin concentrations, but also with increased incidence of febrile episodes in Ghanaian children (50, 51). High ratios of TNFα to IL-10 have also been linked to severe malaria in children from this region (48).

These studies indicate that excessive pro-inflammatory cytokine responses to human malaria infections correlate with more severe clinical symptoms but better parasite clearance. In concert with this evidence, several studies suggest that overproduction of regulatory cytokines has a negative effect on parasite clearance. A 2005 study by Walther et al. revealed that excessive production of anti-inflammatory cytokines such as TGF-β and IL-10 early in infection is linked to reduced ability to control parasite growth (39), and a 2006 study by Prakash et al. indicated that regulatory cytokines were upregulated in patients with cerebral malaria (40). It is interesting to note, however, that this study also found an association between high levels of TNFα, but not IFN-γ, and the development of cerebral malaria (40). This perhaps suggests that classing cytokines as simply pro- or anti-inflammatory, and therefore “good” or “bad”, may be too simplistic when investigating the antimalarial immune response. While clinical immunity to malaria in both mice and humans (defined here as clearance of parasites in the absence of overt clinical symptoms) does seem to require a precise balance between early pro-inflammatory responses needed to kill parasites and regulatory anti-inflammatory responses needed to prevent immune pathology, increasing evidence suggests that the equilibrium between the two is highly complex. For example, Walther et al. noted an association between a higher frequency of FOXP3^−^ CD4^+^ IFN-γ^+^ IL-10^+^ effector T cells in the peripheral blood of children with uncomplicated compared to severe clinical malaria (100), while Jagannathan et al. identified an increased risk of future episodes of malaria in individuals with this same population of FOXP3^−^ CD4^+^ IFN-γ^+^ IL-10^+^ T cells (101). Clearly, the immune response to malaria requires a far more complex investigation than simply stating which cytokines are produced and which are not, and the specific cellular sources of cytokines, the quantity produced, and the timing of their production relative to the time course of infection appear to be key determinants of the outcome of infection.

For a long time, it was assumed that classical αβ CD4 and CD8 T cells were the primary source of malaria-induced IFN-γ as a result of studies where lymphocyte proliferation and IFN-γ production were measured utilizing techniques that could not differentiate between T cells and NK cells (102–104). In more recent times, the advent of intracellular cytokine staining and consequent single cell analysis of cytokine production by flow cytometry has revealed considerable redundancy in the cellular sources of IFN-γ, with γδ T cells and NK cells also producing IFN-γ in response to malaria-infected erythrocytes (32, 105, 106). However, while many different lymphocyte populations are capable of producing IFN-γ, they vary in their relative contributions to the overall IFN-γ response at different stages of infection. The exact proportion of the cytokine response ascribed to NK cells or T cells appears to vary based on the time point examined [reviewed in Ref. (96)] and thus inconsistencies in the literature regarding the major sources of IFN-γ among PBMCs exposed to infected erythrocytes likely reflect differing experimental conditions and differences in the time points chosen for analysis. Very few studies have attempted to establish the full range of cellular sources of IFN-γ over the course of infection (82, 107) [reviewed in Ref. (108)]. In particular, few studies have assessed IFN-γ production in the first 18 h of exposure, and we have shown that assessing IFN-γ production from 24 h coculture onward risks missing important earlier contributions from NK cells (63, 106).

A function for NK cells as early responders in human malaria infection was first suggested in the early 1980s (109), and since that time an increasing number of studies have investigated the role of NK cells in the human antimalarial response. Most of these data are from *in vitro* studies culturing PBMCs or purified NK cells with *P. falciparum*-infected erythrocytes, although a few studies have investigated IFN-γ production and cytotoxic responses *ex vivo* among infected individuals (25, 110–112). *In vitro* studies of PBMCs from malaria-naive individuals have shown that *P. falciparum-*infected erythrocytes can induce NK cells to produce IFN-γ within 6 h of coculture (32), though this response appears to be somewhat heterogeneous between individuals (113). This heterogeneity may be partly explained by NK cell receptor repertoires (114), as evidence suggests that this response may require direct physical contact between NK cells and infected erythrocytes (51, 106). The role of NK cell receptors in determining the magnitude of the IFN-γ response is also supported by evidence from mouse models (83, 115, 116). Optimal NK cell activation also appears to require IL-12 and IL-18 produced by myeloid accessory cells (106), IL-2 from memory CD4^+^ T cells (63), and physical contacts between NK cells and myeloid accessory cells (42, 117, 118), the molecular basis for which has yet to be fully identified. The requirement for IL-12 and IL-2 for optimal NK cell IFN-γ production may explain the association between production of these cytokines and improved clinical outcome in human *P. falciparum* infections (40).

There are many potential *trans* costimulatory signals that could be provided by myeloid accessory cells activated after exposure to infected erythrocytes [reviewed in Ref. (119)], but so far a role has only been demonstrated for interactions between the adhesion molecules ICAM-1 and LFA-1 (118). The role of DCs in human malaria is less clear, but they appear to contribute to NK cell activation by early cytokine production, possibly after activation by parasitized erythrocytes *via* the CD36 scavenger receptor (42, 120). pDCs are a major contributor of type I IFNs in humans during infection with other pathogens due to expression of TLR7 and TLR9 (121) and are hypothesized to play a similar role in malaria infection (120); for example, mouse models support a role for pDCs in NK cell activation during acute infection (67, 71, 72, 122). Similarly, our unpublished work has shown that *in vitro* NK cell responses to *P. falciparum*-infected red blood cells are enhanced by low levels of IL-15, consistent with data from mouse models (74); IL-15 is likely to be *trans*-presented by DCs [reviewed in Ref. (123)].

Recent evidence from mice for the development of “adaptive” or “memory-like” NK cell responses after infection, antigen sensitization, or exposure to inflammatory cytokines (15, 124) is now beginning to be supported by similar (though currently limited) data from human studies (10, 19). NK cells have been shown to contribute to increased IFN-γ responses to malaria antigens after vaccination (26, 98, 99, 125), although initial studies suggest this may be a proxy effect due to priming of antigen-specific CD4 T cells to secrete IL-2 rather than a reflection of intrinsic changes within the NK cell population itself (63). Notably, enhanced IFN-γ production by NK cells from individuals experimentally infected with malaria up to 20 weeks after initial infection appears to be dependent on the presence of both IL-2 and T cells (25).

## NK Cell Cytotoxicity Against Malaria

In addition to the well-established role for NK cells in cytokine production in response to infected erythrocytes, there is limited but growing evidence to suggest that NK cells may also be capable of directly killing *Plasmodium*-infected cells through cytotoxic activity. Cytotoxic granzymes have been detected in the plasma of people with blood-stage malaria infections (111), suggesting that parasite-infected erythrocytes may be targets of NK cell or CD8^+^ T cell cytotoxic activity *in vivo*. Peripheral blood NK cells from experimentally infected malaria-naive volunteers and naturally infected Cameroonian children have also been shown to release cytotoxic mediators when cultured *in vitro* with hepatocytes infected with liver-stage *Plasmodium* (111), with a similar result reported in Kenyan adults and children in response to infected erythrocytes (126). NK cell cytotoxicity has similarly been observed against hepatic-stage parasites in mice (66).

**Figure 2:**
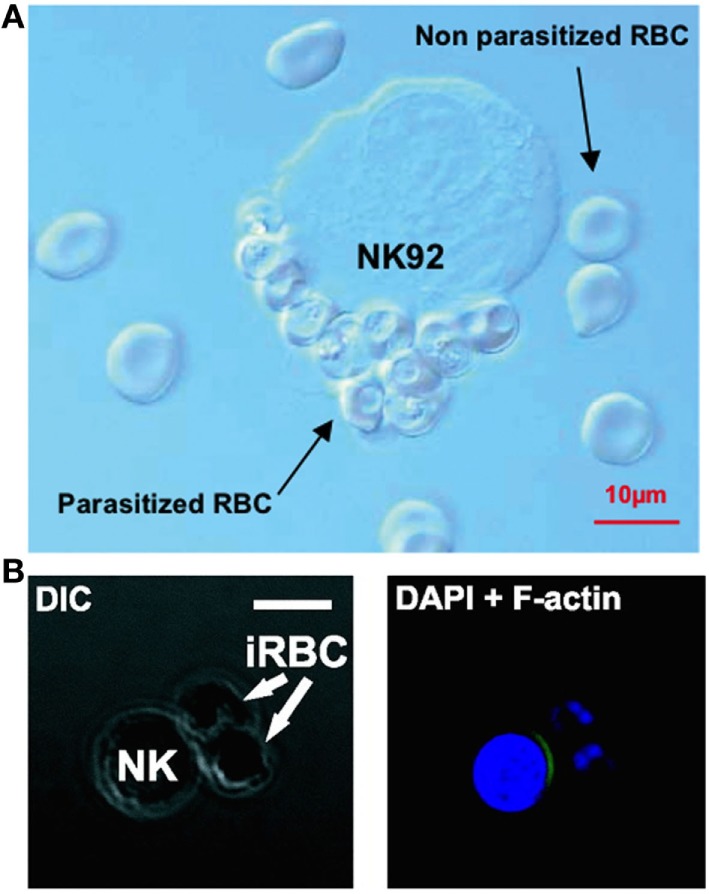
**Evidence of natural killer (NK) cells conjugating to malaria-infected erythrocytes**. NK cells selectively form conjugates or “rosettes” with infected, but not uninfected, erythrocytes. Baratin et al. observed conjugate formation between the NK92 cell line and *P. falciparum*-infected erythrocytes by light microscopy **(A)**. [Figure from Baratin et al. (118). Copyright under Creative Commons license CC-BY.] Korbel et al. observed conjugate formation *via* confocal microscopy **(B)** where NK cells from some donors showed actin relocalization to the site of contact with *P. falciparum*-infected erythrocytes, possibly indicating formation of an immune synapse. [Figure used with permission from Korbel et al. (113). Copyright 2005. The American Association of Immunologists, Inc.]

Natural killer cells have also repeatedly been observed forming stable conjugates with infected erythrocytes *in vitro* (32, 113, 117) (Figure 2). In 2005, Baratin and colleagues found that immortalized NK92 cells selectively bound to infected, but not uninfected, erythrocytes (117) (Figure 2A), while Artavanis-Tsakonas et al. (106) and Korbel et al. (113) observed conjugate formation between *P. falciparum*-infected erythrocytes and freshly isolated human NK cells from multiple individuals (Figure 2B). These conjugates can be observed by light microscopy and can be counted by flow cytometry as singlet events that stain for both NK cell markers and erythrocyte membrane markers (106); these results have been replicated by our group using both flow cytometry markers and a transgenic *P. falciparum* strain expressing green fluorescent protein (127).

Conjugates have been shown to form rapidly (within 30 min), although the proportion of NK cells forming conjugates varies considerably between donors (106, 113), suggesting that the receptors involved in recognition of infected erythrocytes are either polymorphic or are variably expressed on human NK cells. Additionally, in cells from some individuals there is evidence of NK cell cytoskeletal actin rearrangement at the point of contact with infected erythrocytes (113) (Figure 2B). Actin rearrangement at the immune synapse between an NK cell or CD8^+^ T cell and target cells is a prelude to migration of cytotoxic granules toward a target cell (128); as such, these data suggest the formation of a functional cytotoxic synapse between NK cells and infected erythrocytes. More recently, in a humanized mouse model capable of sustaining a *P. falciparum* infection, Chen et al. showed that parasitemia was significantly reduced in the presence of NK cells, and NK cells were directly observed interacting with and killing *P. falciparum*-infected erythrocytes using video microscopy, providing the clearest evidence yet of NK cell cytotoxicity against malaria-infected cells (129).

While there is growing evidence for cytotoxic killing of parasitized cells by NK cells and for functional physical interactions between them, the NK cell receptors and ligands on infected erythrocytes that may mediate these interactions are unknown. Healthy erythrocytes are not known to express either classical or non-classical HLA class I molecules that might represent ligands for inhibitory NK cell receptors such as KIRs or NKG2A, nor are they known to express any known ligands for activating NK cell receptors such as the NCRs. Healthy erythrocytes are therefore generally assumed to go unnoticed by NK cells. However, malaria infection induces numerous perturbations of the erythrocyte membrane [reviewed in Ref. (130)], which may result in the presentation of as yet undiscovered ligands for NK cell receptors.

One parasite-derived molecule that has been widely implicated in interactions between infected erythrocytes and other host cells (and is thus a prime candidate for mediating NK cell interactions with infected erythrocytes) is *Plasmodium falciparum* erythrocyte membrane protein 1 (*Pf*EMP1). *Pf*EMP1 can bind to the adhesion molecules ICAM-1, PECAM, and VCAM, the scavenger receptor CD36, and chondroitin sulfate A (CSA), a glycosaminoglycan modification of many cell surface proteins including those on NK cells (131–133) [reviewed in Ref. (134)]. Polymorphic variants of *Pf*EMP1 display different avidities for these various ligands [reviewed in Ref. (134)]. Baratin et al. (118) have shown that binding of *Pf*EMP1 to CSA mediates binding of infected erythrocytes to NK92 cells, but that this interaction is not required for subsequent activation of the NK cells. In 2007, Mavoungou et al. suggested that *Pf*EMP1 binds to NKp30, a member of the NCR family (135), but a subsequent study by Chen et al. indicated that none of the NCRs (NKp30, NKp44, or NKp46), nor the unrelated activating receptor NKG2D, are required for conjugate formation with infected erythrocytes (129). CD36 expressed on DCs may mediate DC activation by infected erythrocytes (120), but CD36–*Pf*EMP1 interactions have not been shown to occur on NK cells.

Alternatively, a study by Böttger et al. proposed that the presence of membrane-bound heat shock protein 70 (a “self” stress ligand) on the surface of infected erythrocytes may be sufficient to trigger release of granzyme B from NK cells, leading to subsequent erythrocyte death (136); however, this has not been subsequently confirmed. Other studies have demonstrated that, when compared to live and intact infected erythrocytes, dead or lysed infected erythrocytes do not fully activate NK cells (32, 51), which may narrow down possible candidate ligands, although it is possible that this simply reflects a reduced capacity of dead parasite material to fully activate myeloid accessory cells or IL-2-producing CD4^+^ T cells rather than evidence of a reduced capacity to bind NK cells directly.

## Heterogeneity in NK Cell Responses to Malaria

*In vitro*, NK cell responses to infected erythrocytes differ greatly between individuals, although individuals’ responses are consistent over time (106, 113, 114). This diversity likely arises from a number of sources including the strength of the cytokine and costimulatory signals provided to NK cells by myeloid accessory cells (42), differential NK cell maturation status dependent on age and infection with human cytomegalovirus (137) [reviewed in Ref. (138)], frequencies of malaria-reactive or cross-reactive IL-2-secreting CD4^+^ T cells (25, 63, 98, 99), and genetically determined differences in the expression of NK cell activating and inhibitory receptors, which set the threshold for NK cell activation (7, 114) [reviewed in Ref. (139)]. Among these, there is evidence that genetic diversity genetic diversity in KIRs may contribute to the heterogeneity of the response to infected erythrocytes.

The KIR locus contains genes for both activating (short-tailed) and inhibitory (long-tailed) KIR, and heterogeneity in gene content combined with allelic polymorphism at individual loci and stochastic expression of individual receptors at the cellular level leads to extensive haplotypic diversity and highly diverse NK cell populations within an individual (140). There are two distinct families of KIR haplotypes, comprising combinations of KIRs commonly inherited together. The A haplotype encodes mainly inhibitory receptors with KIR2DS4 the only activating receptor, while the B haplotype encodes more balanced combinations of inhibitory and activating receptors (141) [reviewed in Ref. (142, 143)]. However, genes in the centromeric and telomeric regions of the two haplotypes can recombine during meiosis, leading to hybrid centromeric A/telomeric B haplotypes and *vice versa* (144, 145) [reviewed in Ref. (29)].

Heterozygous carriage of the AB KIR haplotypes appears to be associated with increased IFN-γ production *in vitro* in response to iRBC compared to either AA or BB homozygous individuals (114). Similarly, AB heterozygosity was suggested to be protective during clinical malaria infection, as individuals carrying c-AB2/t-AA (i.e., individuals with heterozygous A and B centromeric KIR genes in combination with telomeric A haplotype genes) were more likely to have asymptomatic malaria infections rather than uncomplicated or severe symptomatic malaria (146). As carriage of both A and B haplotypes is likely to increase the total number of different KIR that can be an expressed by an individual, heterozygosity may increase the proportion of NK cells that express a KIR capable of binding self-HLA and are therefore “licensed” [reviewed in Ref. (28, 147)] and, as such, may be more responsive to activation by pathogens. Conversely, in a Gambian population, Yindom et al. suggested that an AA KIR haplotype may be protective during malaria infection and that carriage of activating KIRs is associated with higher mortality (148); this may suggest that NK cell responses contribute to the over-exuberant inflammatory responses that are associated with severe disease, either because they express a particular activating KIR that recognizes a, as yet unknown, ligand on infected erythrocytes or because the balance of activating to inhibitory KIR expressed by B haplotype-bearing NK cells lowers the threshold for activation [reviewed in Ref. (149)]. To date, the largest genetic association study of KIR and malaria susceptibility, conducted in Thailand, reported that KIR2DL3 in association with its ligand HLA-C1 is associated with an increased risk of cerebral malaria compared to uncomplicated malaria and that this combination of KIR2DL3–HLA-C1 is significantly less common in malaria-endemic areas than might be expected; the authors proposed that this was evidence of natural selection (150). One hypothesis that might unify all of these findings is that protection is mediated by KIR2DL2, a B haplotype KIR, which binds HLA-C1 with higher affinity than KIR2DL3, an A haplotype KIR (151); thus, carriage of a single copy of the centromeric B haplotype may confer protection against severe malaria by preventing interactions between KIR2DL3 and HLA-C1 through preferential expression of KIR2DL2. However, larger studies of KIRs that take into account the genetic background of the population and the allelic diversity of both KIR and HLA class I molecules are needed to determine whether KIR receptors do in fact influence malaria disease progression. In this respect, it is disappointing that all of the recent genome-wide association studies of malaria susceptibility exclude KIR and/or HLA from their analyses (152, 153).

A major limitation of NK cell licensing as a possible explanation for the association between KIR AB heterozygosity and resistance to malaria is that licensing has only been shown to enhance NK cell cytotoxicity in situations where MHC class I expression is downregulated (i.e., missing self) [reviewed in Ref. (139)]. For activation *via* missing self, at least in terms of lack of MHC class I, to operate in malaria infections, the balance of NK cell receptor signaling would probably have to be altered by expression of a potent activating ligand. Moreover, it is not clear that licensed NK cells display a greater capacity for cytokine production (which is the more established role for NK cells in malaria infection) compared to unlicensed cells (154). Indeed, CD56^dim^ NK cells (which express KIR) seem to produce lower levels of IFN-γ than CD56^bright^ NK cells that lack KIR expression (106, 114), and in some viral infections unlicensed cells are thought to be key producers of cytokines (155). Finally, in considering the potential role of KIR in controlling NK cell responses to malaria, more definitive evidence is required for direct, functional interactions between NK cells and infected erythrocytes, and it is also necessary to consider that genetic associations between malaria severity and KIR might be mediated by NK cell interactions with infected hepatocytes (which express MHC class I) during the pre-erythrocytic liver stage of infection.

## Concluding Comments

**Figure 3:**
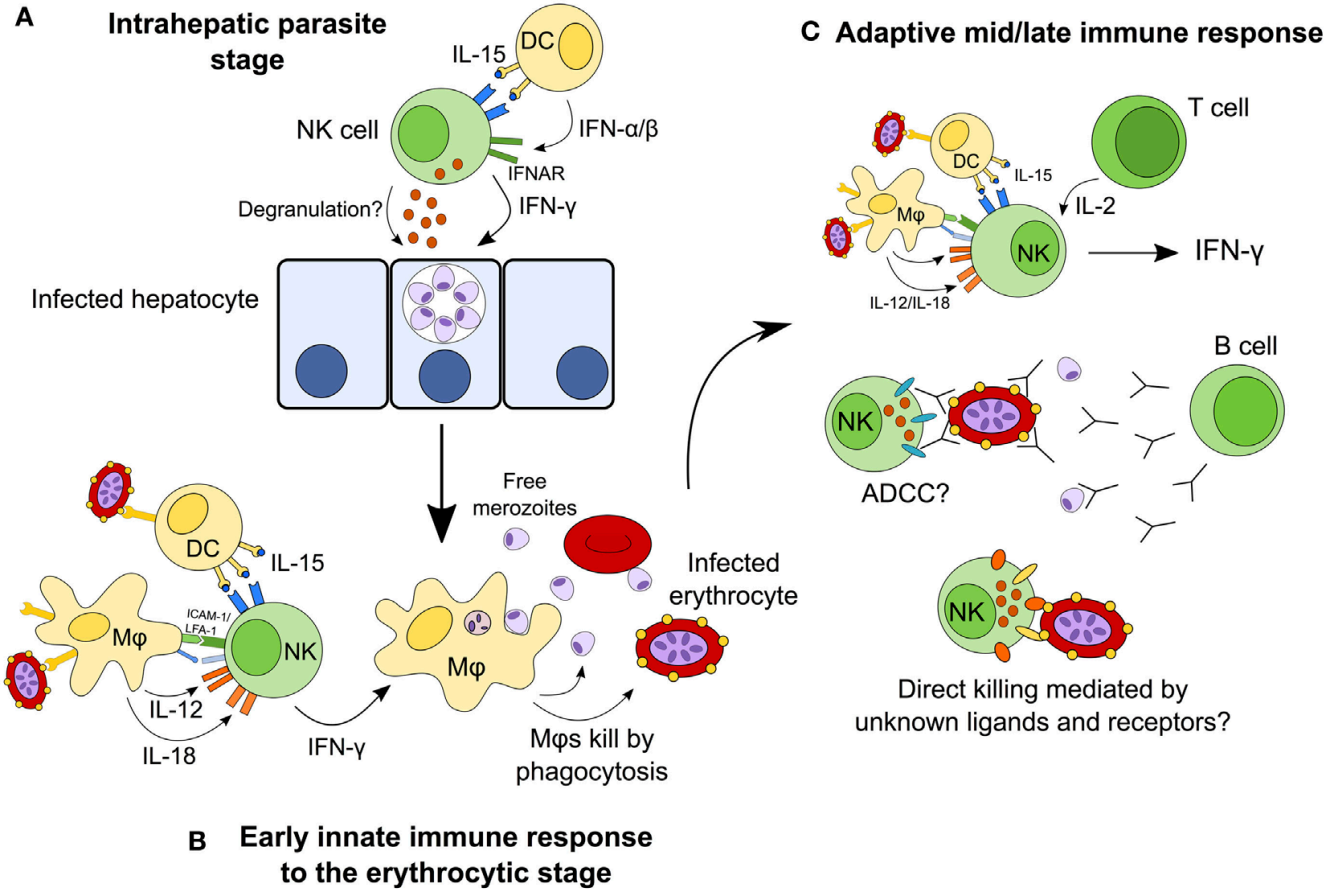
**Hypothesized model for the continuing role of natural killer (NK) cells during malaria infection**. NK cells may destroy infected hepatocytes by perforin/granzyme-mediated cytotoxic killing or death receptor-induced apoptosis, or may kill the parasite within the hepatocyte *via* cytokine-mediated induction of toxic radicals **(A)**. During the early erythrocytic stage of infection, NK cells are activated by cytokines from macrophages and dendritic cells (DCs) and in turn release interferon gamma (IFN-γ) to activate macrophages that phagocytose infected erythrocytes **(B)**. Once the adaptive immune response has developed, T cells contribute interleukin (IL)-2 to enhance the ongoing NK cell response. In the presence of specific IgG antibodies, NK cells may now mediate parasite clearance and killing by antibody-dependent cell-mediated cytotoxicity (ADCC). NK cells may also kill infected erythrocytes directly *via* formation of an immune synapse and release of cytolytic granules **(C)**.

Natural killer cells have traditionally been considered to contribute to the control of infection by producing IFN-γ and killing infected cells during the first hours and days of infection, before being superseded by the adaptive immune response. This narrow interpretation of NK cells as mediators of innate immunity has had to be re-evaluated in light of more recent studies implicating NK cells as effectors in the adaptive immune response (mediating antibody-dependent cellular cytotoxicity and responding to IL-2 from effector and effector memory CD4^+^ T cells) (19, 24, 25) [reviewed in Ref. (9, 156)]. In the case of malaria, NK cells have been implicated in cytokine-mediated as well as cytotoxic activity against both erythrocyte and liver-stage parasites, and in both early and late stages of infection. Although the currently available data tend to support a primarily beneficial role for NK cells as an early source of a key cytokine (IFN-γ) and suggest that they might also contribute to controlling parasitemia by lysis of infected erythrocytes, these studies fall far short of convincingly demonstrating either a protective or deleterious role for NK cells in human malaria infection. Figure 3 shows the different roles that NK cells may play throughout the course of malaria infection; future studies may confirm or refute these suggestions. An alternate hypothesis is that, in some people, NK cell cytokine production may contribute to immune pathology. If so, this may be a transient effect associated with particular stages of the development of antimalarial immunity.

Interferon gamma is produced by many lymphocyte populations at the various stages of malaria infections (including αβ CD4^+^ T cells, γδ T cells, and NK T cells (112)), suggesting that the particular importance of NK cells as cytokine producing cells is during the very first few days of a blood-stage infection (63). The role of NK cells later in malaria infection, or upon secondary or subsequent infection, and in particular the importance of NK cell-mediated cytotoxicity, ADCC and NK cell “memory,” requires further investigation. As we learn more about how KIR and HLA genotypes influence NK cell function and licensing, and how NK cell phenotype and function change over the course of life, we may gain a better understanding of the role of NK cells throughout the course of malaria infections.

## Author Contributions

This review was written jointly by A-SW, SS, and EM.

## Conflict of Interest Statement

The authors declare that the research was conducted in the absence of any commercial or financial relationships that could be construed as a potential conflict of interest.
